# Gene expression analysis of overwintering mountain pine beetle larvae suggests multiple systems involved in overwintering stress, cold hardiness, and preparation for spring development

**DOI:** 10.7717/peerj.2109

**Published:** 2016-07-06

**Authors:** Jeanne A. Robert, Tiffany Bonnett, Caitlin Pitt, Luke J. Spooner, Jordie Fraser, Macaire M.S. Yuen, Christopher I. Keeling, Jörg Bohlmann, Dezene P.W. Huber

**Affiliations:** 1 Department of Ecosystem Science and Management, University of Northern British Columbia, Prince George,British Columbia,Canada; 2 Department of Michael Smith Laboratories, University of British Columbia,Vancouver,British Columbia,Canada; 3 Department of Biological Sciences, Simon Fraser University,Burnaby,British Columbia,Canada

**Keywords:** Cold tolerance, Mountain pine beetle, Insect overwintering, Glycerol, Physiological stress, Lodgepole pine, Heat shock proteins, Anti-freeze, Cytochromes P450

## Abstract

Cold-induced mortality has historically been a key aspect of mountain pine beetle, *Dendroctonus ponderosae* Hopkins (Coleoptera: Curculionidae), population control, but little is known about the molecular basis for cold tolerance in this insect. We used RNA-seq analysis to monitor gene expression patterns of mountain pine beetle larvae at four time points during their overwintering period—early-autumn, late-autumn, early-spring, and late-spring. Changing transcript profiles over the winter indicates a multipronged physiological response from larvae that is broadly characterized by gene transcripts involved in insect immune responses and detoxification during the autumn. In the spring, although transcripts associated with developmental process are present, there was no particular biological process dominating the transcriptome.

## Introduction

Changing climate and large contiguous stands of susceptible lodgepole pine (*Pinus contorta* Douglas) have resulted in a large mountain pine beetle, *Dendroctonus ponderosae* Hopkins (Coleoptera: Curculionidae) outbreak in British Columbia and other parts of western North America (Raffa et al., 2008). The beetle and its fungal associates are projected to kill an estimated 750 million cubic meters of merchantable pine in British Columbia by 2017 (Natural Resources Canada, 2015). The large scale of this outbreak in British Columbia, in combination with the expansion of mountain pine beetle into previously unaffected areas of jack pine, *Pinus bankisana* Lamb, forests in Alberta (Cullingham et al., 2011), have caused substantial economic losses in British Columbia (Schneider et al., 2010) and continue to have massive ecological effects on the landscape.

Cold-induced mortality has historically been a key aspect of mountain pine beetle population control. The mountain pine beetle spends much of its typically one-year life cycle as larvae in the phloem tissue of susceptible host trees. While they overwinter under the bark, larvae have had, under typical conditions, to survive winter temperatures below –30°C (Cole, 1981). Mountain pine beetle larvae possess cold tolerance mechanisms, such as the production of glycerol (Bentz & Mullins, 1999) that enable supercooling of insect bodily fluids and tolerance of temperatures far below freezing. However, if a cold snap occurs early in winter, before the larvae have become cold acclimated, massive mortality can occur (Bale, 2002). Cold-induced mortality has historically controlled bark beetle populations in British Columbia and prevented the insects from moving further north or east than their historically known range. Because of the recent move of this insect across the Rocky Mountains and into the jack pine forests of Alberta (Cullingham et al., 2011; Janes et al., 2014), understanding the cold tolerance mechanisms of mountain pine beetle is becoming increasingly important for forest management and the development of predictive models.

We used RNA-seq analysis to monitor transcript profiles of mountain pine beetle larvae at four time points during their overwintering period—early-autumn, late-autumn, early-spring, and late-spring. Changing transcript profiles over the winter indicates a multipronged approach to cold readiness, overwintering, and transition into spring development in mountain pine beetle larvae. We uncovered shifts in transcript levels for several groups of genes that are likely to be important in the overwintering success of mountain pine beetle larvae.

## Methods and Materials

### Collection of larval specimens

Overwintering larvae were collected as described in Bonnett et al., 2012. In brief, larvae were sampled from eleven naturally infested lodgepole pine trees located at two sampling sites near Tête Jaune Cache, British Columbia, Canada (N53°3′36.00″, W119°36′54.00″and N52°55′4.00″, W119°21′23.00″). Each tree was fitted with three iButton temperature data loggers (Maxim, Sunnyvale, CA) that recorded ambient temperature every thirty minutes. Overwintering mountain pine beetle larvae were live-collected from under the bark, immediately flash frozen with liquid nitrogen in individual vials in the field, transported on dry ice, and stored at –80°C until RNA extractions were conducted. Collection dates were 26 September 2008, 7 November 2008, 25 March 2009, and 27 May 2009.

### RNA extractions

Immediately prior to RNA extractions, larval beetles were transitioned to –20°C in RNAlater-ICE Frozen Tissue Transition Solution (Ambion, Life Technologies) according to the manufacturer’s protocol, and placed in individual wells of a 96-well, 1 mL round bottom polypropylene block (Corning Life Sciences) with one stainless steel grinding ball per well. RNA extractions were performed using the MagMAX-96 Total RNA Isolation Kit (Ambion, Life Technologies). Prior to extraction, RNAlater-ICE solution was removed by pipette, and 100 µL of lysis/binding solution from the MagMax-96 Total RNA Isolation Kit was immediately added to each sample well. Samples were ground for eight cycles (1500 strokes/min for 30 s followed by 30 s on ice) using a Geno/Grinder 2000 (SPEX CertiPrep). Instructions for the MagMax-96 Total RNA Isolation Kit were followed with a few minor modifications: when initially transferring samples from the grinding plate to the processing plate, the 60 µL of isopropanol was added directly to the wells in the grinding plate to aid with sample transfer; during the DNA digestion step, 60 µL of diluted TURBO DNase was added to each sample to ensure complete DNA degradation; before the final elution step, the elution buffer was heated to 70°C to ensure maximum bead dispersion, as suggested in the troubleshooting guide. RNA isolates from individual larvae were assessed for concentration and purity on a Nanodrop-1000 Spectrophotometer (Thermo Fisher Scientific).

### RNA sample pooling and replicates

For the RNA-seq analysis, we used four biological replicates for each of the four sampling dates. Due to the small size of the larvae, each ‘biological’ replicate was comprised of pooled individual larvae. For the small September larvae, up to eight RNA extractions from individual larvae were pooled to get a single biological replicate. For the November, March, and May larvae, four individuals were pooled for each biological replicate. Pooled RNA samples were concentrated using the RNeasy Mini Kit (Qiagen), following the instructions for the RNA Cleanup Protocol. An aliquot of purified RNA for each biological replicate, containing from 5–11 µg of RNA in a maximum volume of 100 µL, was submitted to the Canada’s Michael Smith Genome Sciences Centre (Vancouver, BC, Canada) for whole transcriptome shotgun sequencing.

### RNA-seq method

Paired-end Illumina HiSeq 2000 platform sequencing was performed as described in Robert et al. (2013). 50 bp sequence reads were requested, although 75 bp reads were generated for some of the sequencing lanes because of advancing sequencing technologies. The 16 samples were multiplexed into four sequencing lanes, with one replicate of each treatment randomly assigned per lane so that every lane contained all four sampling dates. The paired-end fastq files for each library were mapped to the 13,088 gene models identified in the male mountain pine beetle genome sequence (Keeling et al., 2013) via CLC Genomics Workbench (CLC bio: http://www.clcbio.com/) using the parameters listed in Robert et al. (2013). The average fragment length for paired-end reads was approximately 220bp. Read counts are used as a measure of transcript expression for a given gene model in each sample.

The raw RNA-seq data is available at the National Center for Biotechnology Information Sequence Read Archive (NCBI SRA) database (accession numbers: SRX1043704–SRX1043719) under the TRIA umbrella BioProject (PRJNA169907) that identifies aggregated research project data generated from the TRIA research collaborations on mountain pine beetle systems genomics.

DESeq statistical analysis based on Anders & Huber (2010) was also performed as described in Robert et al. (2013) to calculate fold-changes for each gene as well as *p*-values adjusted (padj) for multiple comparisons using a Benjamini–Hochberg correction (Benjamini & Hochberg, 1995). We used a 0.1% false discovery rate (padj < 0.001) to identify transcript expression levels that were significantly different between timepoints. The DESeq output is publicly available via a DOI-based repository and can be found in ‘Data Availability’.

## Results

### Sequencing results

**Figure 1:**
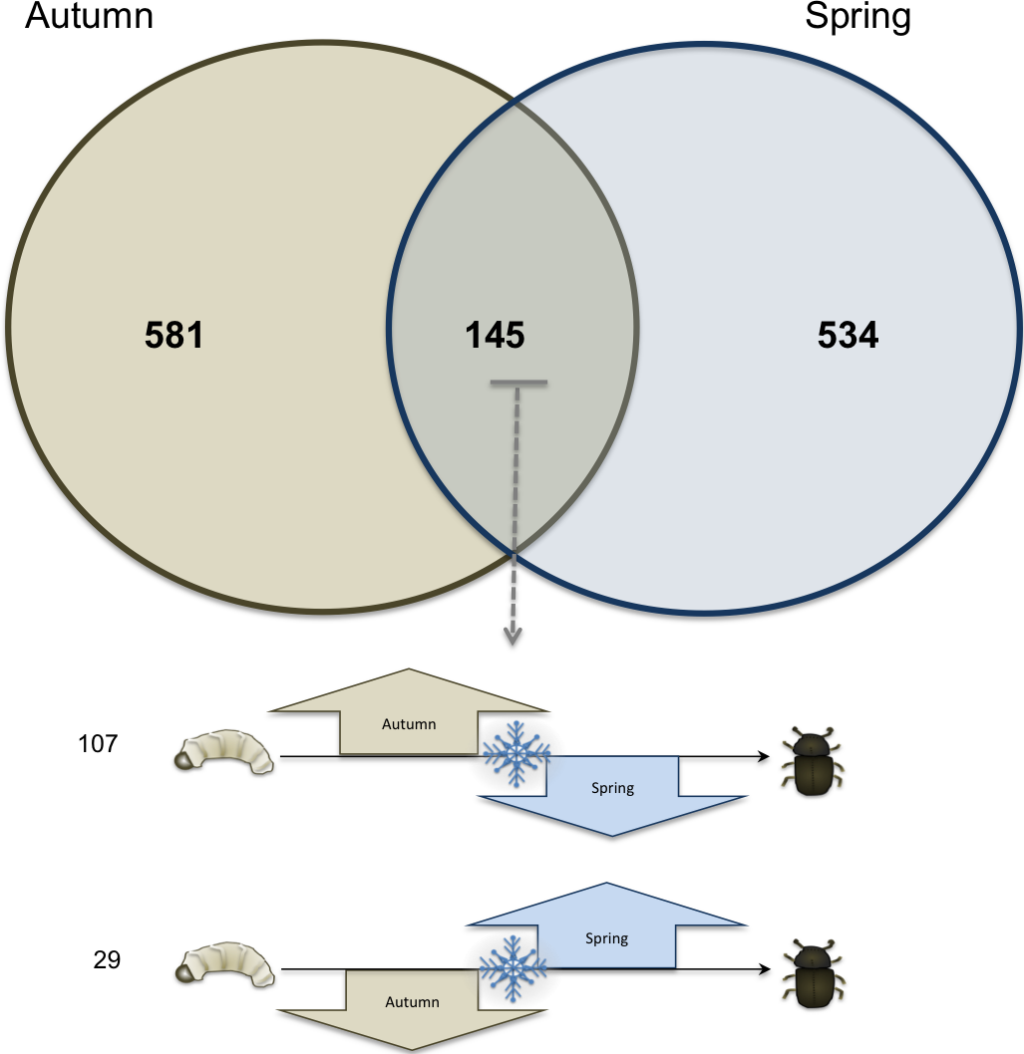
Venn diagram of total transcriptome. Venn diagram showing the number of significantly changing transcripts unique to autumn (brown circle), and unique to spring (blue circle), and those that showed significantly shifts in both autumn and spring (middle). Of the 145 gene transcripts that showed a significant difference in both autumn and spring, 107 increased in autumn and decreased in spring, and 29 are decreased in autumn and then increased in spring.

Sixteen libraries were sequenced with four biological replicates at each time point (Table 1). Overall, 581 transcripts (representing 4.43% of the 13,088 gene models, or predicted genes, identified in the draft mountain pine beetle genome) with significant shifts in expression occurred uniquely between the time points in September and November (Autumn) and 534 (representing 4.08% of the gene models identified in the draft genome) occurred uniquely between the time points in March to May (Spring); 145 transcripts (representing 1.12% of the gene models in the draft genome) showed significantly shifts in both the fall and winter (Fig. 1). Of the shared 145 transcripts, 107 increased in autumn and decreased in the spring whereas 29 transcripts showed the opposite pattern (i.e., decreased in autumn and increased in spring). The remaining nine significantly changing transcripts either both increased in autumn and spring, or both decreased in autumn and spring.

**Table 1:** Summary information for sequence data mapped to the male mountain pine beetle genome (Keeling et al., 2013).

Sampling date	Replicate	NCBI accession number	Read length	Total pairs mapped	Uniquely mapped pairs
Sept. 26, 2008	1	SRX1043704	50	51,228,868	20,383,049
	2	SRX1043705	50	75,540,024	29,715,613
	3	SRX1043706	75	66,662,746	26,832,203
	4	SRX1043707	75	41,552,862	16,802,040
Nov. 7, 2008	1	SRX1043708	50	64,102,946	25,410,259
	2	SRX1043709	50	52,214,600	20,525,750
	3	SRX1043710	75	36,280,228	14,402,268
	4	SRX1043711	75	71,571,952	28,268,130
Mar. 25, 2009	1	SRX1043712	50	59,279,874	23,425,001
	2	SRX1043713	50	54,870,752	21,496,616
	3	SRX1043714	75	58,248,050	23,687,547
	4	SRX1043715	75	67,215,526	26,666,241
May 27, 2009	1	SRX1043716	50	55,731,196	21,959,708
	2	SRX1043717	50	43,999,520	17,182,971
	3	SRX1043718	75	73,662,736	29,422,596
	4	SRX1043719	75	51,860,004	20,696,257

### Transcriptome changes during autumn (September to November)

Between September and November, 726 transcripts showed significantly different expression levels (Bonferroni adjusted *p*-value < 0.001). Of these 726, 458 were significantly up-regulated (ranging from 1.6-fold increase to >100-fold increase) and 268 were significantly down-regulated (ranging from a 1.7-fold decrease to >300-fold decrease).

The largest fold changes that occurred between sampling dates in September and in November were gene transcripts annotated in the broad group of stress response physiology. The top expression fold-change was annotated as a putative galactose-specific C-type lectin (102.75-fold increase), an enzyme associated with the immune response in insects (e.g., Yu et al., 2005). Of the 11 transcripts that were significantly up-regulated over 10-fold between September and November (Table 2): four were of unknown function and were associated with physiological stress arising from insect contact with pathogens and host plant defenses as well as general cellular stabilization responses in response to stress. These six transcripts were annotated as a putative alpha-esterase, glycoside hydrolase family protein 48, cyclin-dependent kinase 5 activator, two heat shock proteins, and a glutathione S-transferase.

**Table 2:** Positive fold changes greater than 10. Differentially expressed transcripts (identified by the gene model ID in the mountain pine beetle draft genome (Keeling et al., 2013) with positive fold changes >10 between September and November, and their annotations.

NCBI accession	Fold change	Adjusted *p*-value	Annotation
KB740939.1	102.7	1.19 × 10^−6^	galactose-specific C-type lectin (*Aedes aegypti*)
KB740085.1	85.9	1.18 × 10^−48^	hypothetical protein (*Tribolium castaneum*)
KB740839.1	19.1	6.43 × 10^−13^	alpha-esterase (*Tribolium castaneum*)
KB740648.1	15.8	4.46 × 10^−12^	hypothetical protein (*Tribolium castaneum*)
KB740914.1	14.2	0.00052	glycoside hydrolase family protein 48 (*Dendroctonus ponderosae*)
KB740085.1	14.1	3.2 × 10^−08^	GF10288 (*Drosophila ananassae*)
KB740939.1	12.7	1.03 × 10^−5^	cyclin-dependent kinase 5 activator (*Tribolium castaneum*)
KB741213.1	11.9	2.25 × 10^−8^	No hits
KB740969.1	11.9	1.19 × 10^−21^	heat shock protein 68 (*Tribolium castaneum*)
KB740969.1	11.1	5.65 × 10^−50^	heat shock protein 68 (*Tribolium castaneum*)
KB741231.1	10.8	2.69 × 10^−7^	putative glutathione s-transferase (*Tribolium castaneum*)

Conversely, of the 41 transcripts that showed the largest (>10-fold) down-regulation between September and November, almost half (19) have no known function, a further nine were annotated as insect cuticle proteins, and the remaining 13 transcripts were variously annotated, but contain a number of catabolic enzymes such as proteases and lyases (Table 3).

**Table 3:** Negative fold changes greater than 10. Differentially expressed transcripts (identified by the gene model ID in the mountain pine beetle draft genome (Keeling et al., 2013) with negative fold changes >10 between September and November, and their annotations.

NCBI accession	Fold change	Adjusted *p*-value	Annotation
KB741103.1	–10.18	0.00022	Pupal cuticle protein
KB741156.1	–10.20	8.08 × 10^−05^	CLIP-domain serine protease (*Tribolium castaneum*)
KB740994.1	–11.16	6.50 × 10^−8^	hypothetical protein (*Tribolium castaneum*)
KB741213.1	–11.30	7.35 × 10^−20^	No hits
KB741217.1	–11.71	0.00048	cytochrome P450 (*Tribolium castaneum*)
KB740848.1	–12.02	4.45 × 10^−5^	cuticle protein (*Tribolium castaneum*)
KB741159.1	–12.41	0.00073	No hits
KB741077.1	–12.48	5.14 × 10^−9^	No hits
KB740085.1	–12.71	4.51 × 10^−5^	hypothetical protein (*Tribolium castaneum*)
KB740993.1	–13.09	4.45 × 10^−5^	cuticle protein (*Tribolium castaneum*)
KB741103.1	–13.52	3.47 × 10^−15^	No hits
KB740939.1	–13.67	7.13 × 10^−5^	PREDICTED: similar to pol (*Hydra magnipapillata*)
KB740540.1	–13.73	3.88 × 10^−15^	hypothetical protein (*Tribolium castaneum*)
KB740970.1	–13.95	1.61 × 10^−18^	polysaccharide lyase (*Dendroctonus ponderosae*)
KB740193.1	–14.26	1.83 × 10^−12^	nicotinic acetylcholine receptor (*Tribolium castaneum*)
KB740085.1	–14.29	3.34 × 10^−8^	hypothetical protein (*Tribolium castaneum*)
KB739553.1	–14.98	8.58 × 10^−5^	acid phosphatase (*Tribolium castaneum*)
KB740193.1	–15.16	9.54 × 10^−7^	nicotinic acetylcholine receptor (*Musca domestica*)
KB741019.1	–16.07	2.20 × 10^−16^	No hits
KB741019.1	–16.85	1.07 × 10^−12^	No hits
KB741269.1	–17.96	1.57 × 10^−5^	hypothetical protein (*Tribolium castaneum*)
KB740727.1	–18.73	0.00022	glucose dehydrogenase (*Tribolium castaneum*)
KB741062.1	–20.00	1.78 × 10^−73^	endopolygalacturonase ( *Dendroctonus ponderosae*)
KB740972.1	–22.23	0.00064	ectodermal protein (*Tribolium castaneum*)
KB741177.1	–23.82	1.02 × 10^−9^	hypothetical protein (*Tribolium castaneum*)
KB741019.1	–26.06	4.58 × 10^−6^	No hits
KB741018.1	–26.81	0.00057	cuticle protein (*Culex quinquefasciatus*)
KB741067.1	–31.69	1.25 × 10^−6^	larval cuticle protein (*Apriona germari*)
KB741207.1	–32.00	0.00012	Hypothetical protein (*Tribolium castaneum*)
KB740798.1	–33.75	0.00026	hypothetical protein (*Tribolium castaneum*)
KB740972.1	–34.89	2.75 × 10^−11^	No hits
KB740425.1	–36.77	0.00037	Hypothetical protein (*Tribolium castaneum*)
KB740686.1	–55.12	0.00056	Hypothetical protein (*Drosophila pseudoobscura pseudoobscura*)
KB740085.1	–70.29	0.00014	cuticular protein (*Tribolium castaneum*)
KB740880.1	–124.19	9.00 × 10^−5^	hypothetical protein (*Tribolium castaneum*)
KB738495.1	–157.19	1.89 × 10^−5^	cuticular protein (*Tribolium castaneum*)
KB740540.1	–181.29	0.00093	hypothetical protein (*Tribolium castaneum*)
KB740085.1	–206.30	8.84 × 10^−5^	cuticular protein (*Tribolium castaneum*)
KB740966.1	–298.81	1.06 × 10^−7^	Hypothetical protein (*Drosophila erecta*)
KB736863.1	–301.59	0.00044	No hits
KB740984.1	–313.04	9.44 × 10^−7^	cuticle protein (*Tribolium castaneum*)

### Transcript changes during spring (March to May)

Between the sampling points in March and May, 679 gene transcripts showed significant differences (Bonferroni adjusted *p*-value < 0.001). The 456 transcripts significantly up-regulated between March and May, as a group, had very different annotations than the transcript changes associated with the autumn period between September and November. The top six transcripts, in terms of fold-changes, between March and May all exhibited well over a 100 fold increase. Of these six transcripts, the greatest up-regulation was >1000-fold increase in a transcript annotated as a cathepsin L-like proteinase, a digestive enzyme in beetles (Murdock et al., 1987). The second highest fold change (202-fold up-regulation) is annotated as a *pol* (polyp-specific) gene similar to a sequence expressed in the polyp stage of the freshwater cnidarian, *Hydra magnipapillata*. The remaining transcripts that exhibited >100-fold up-regulation were all without functional annotations in current databases.

### Patterns in transcript changes during overwintering (September to May)

**Figure 2:**
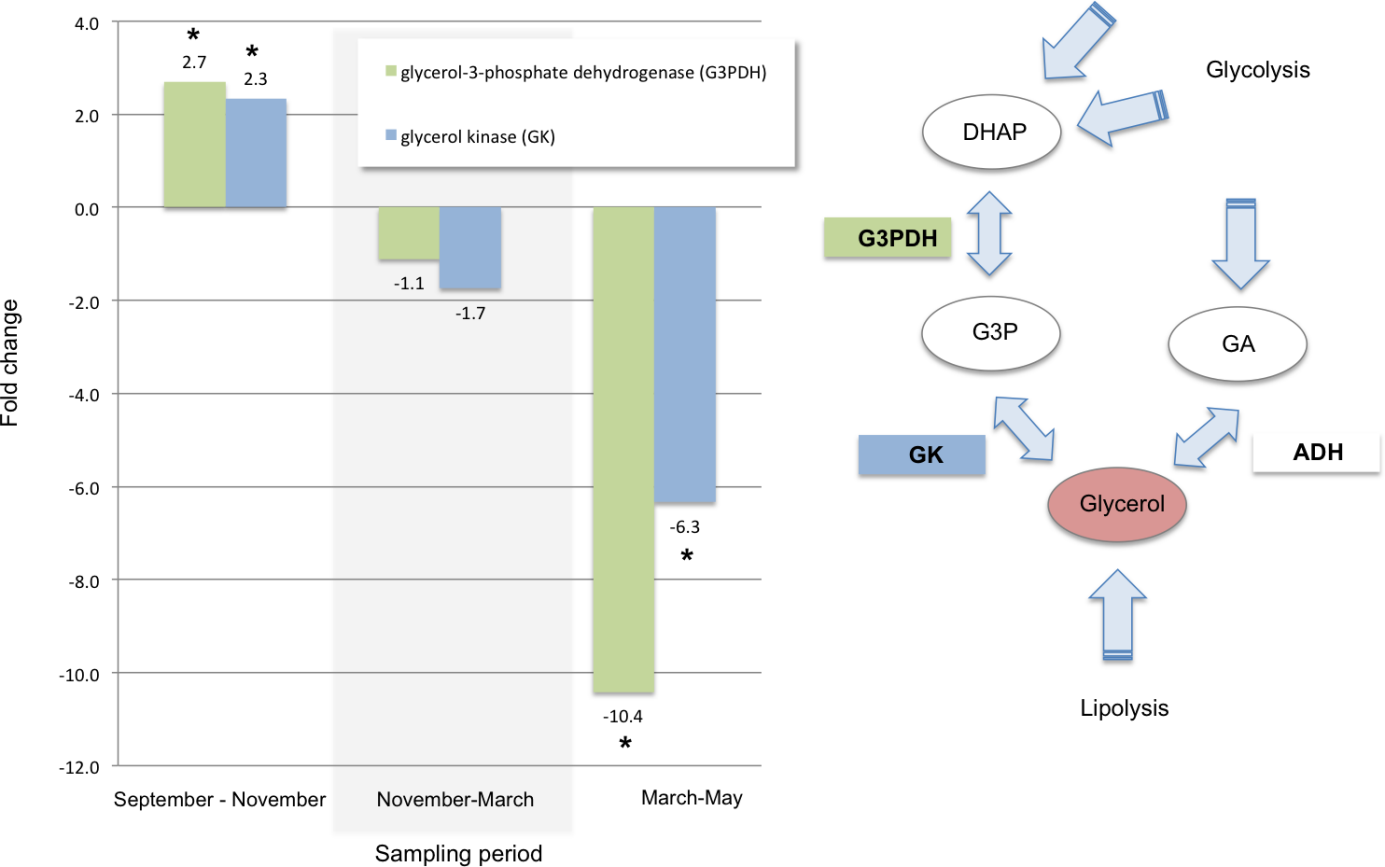
Glycerol biosynthesis. Fold changes between each sampling date through the winter for transcripts annotated as key enzymes in the biosynthetic pathway for glycerol: glycerol-3-phosphate dehydrogenase (G3PDH, KB741112.1) and glycerol kinase (GK, KB740969.1). An asterisk indicates a significant fold change within the two time points measured within each sampling period (Bonferroni adjusted *p*-value < 0.001). The diagram on the right illustrates a subset of the glycerol biosynthetic pathway (modified with permission from Fraser, 2011) showing the location G3PDH and GK as the enzymes that catalyze the steps immediately preceding the production of glycerol. Metabolites are shown in ovals: dihydroxyacetone phosphate (DHAP), glycerol-3-phosphate (G3P), glyceraldehyde (GA); enzymes are shown in rectangles: glycerol-3-phosphate dehydrogenase (G3PDH), glycerol kinase (GK), alcohol dehydrogenase (ADH).

Of the transcripts that exhibited increased levels between September and November, 107 also decreased significantly between March and May. Many of these were annotated as genes responsible for cold tolerance and as indicators of physiological response to stress. Transcripts associated with cold tolerance included those for two enzymes—glycerol-3-phosphate dehydrogenase and glycerol kinase—that immediately precede the production of glycerol. Those two were also significantly up-regulated in the autumn, stable over the winter, and significantly down-regulated in the spring (Fig. 2).

**Figure 3:**
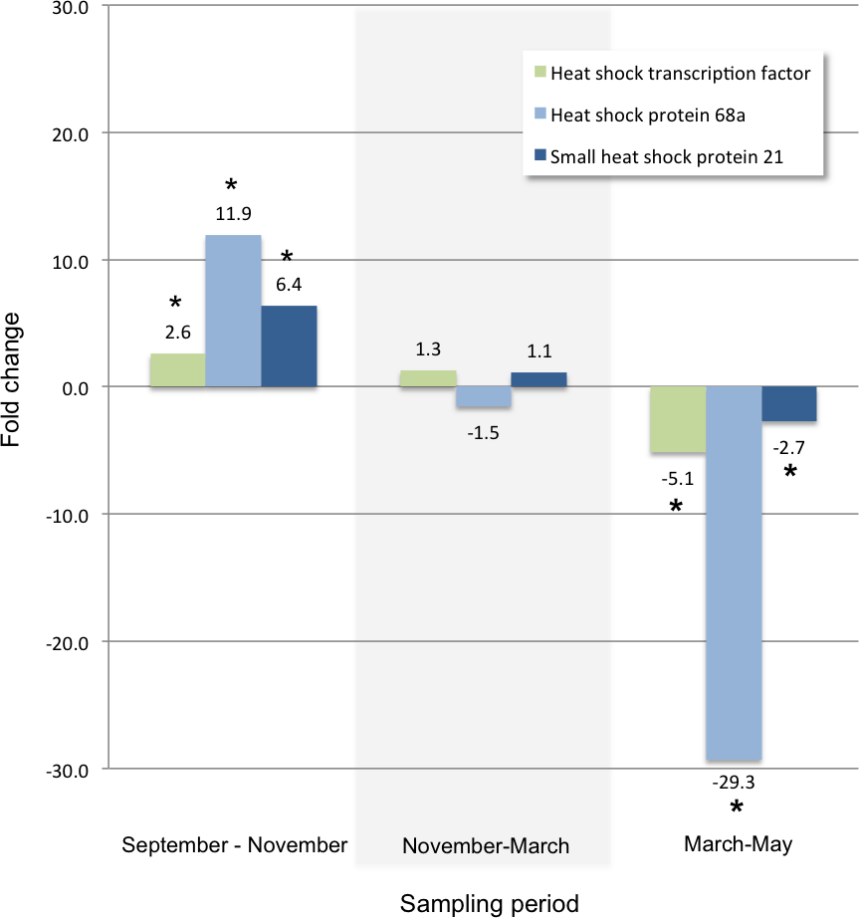
Heat shock proteins. Fold changes between each sampling date through the winter for transcripts annotated with: a heat shock transcription factor (KB740969.1), heat shock protein 68a (KB740941.1), and small heat shock protein 21 (KB740193.1). An asterisk indicates a significant fold change between the time points within each sampling period (Bonferroni adjusted *p*-value < 0.001).

**Figure 4:**
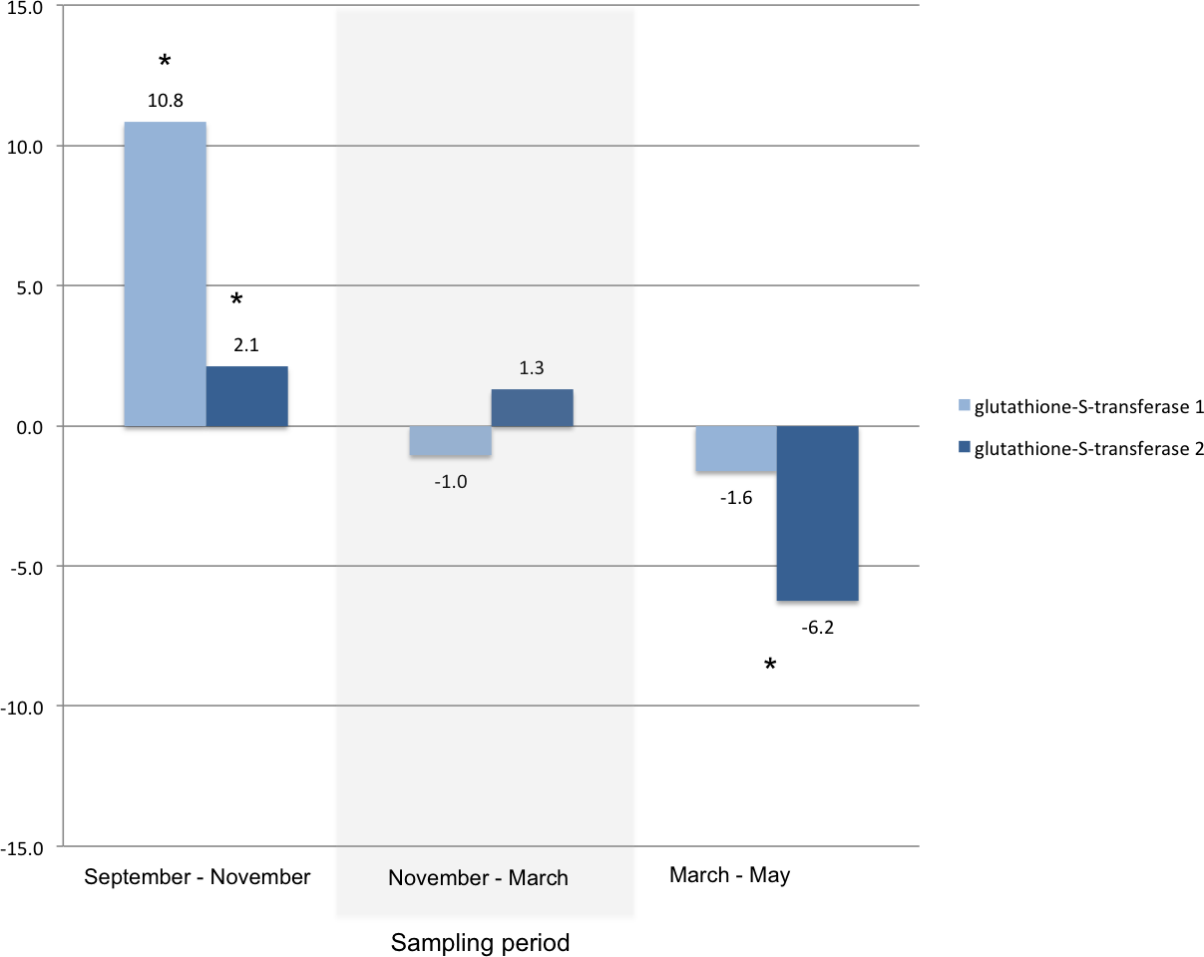
Glutathione-S-transferase. Fold changes between each sampling date through the winter for transcripts annotated as glutathione-S-transferase 1 (KB741231.1) and glutathione-S-transferase 2 (KB740293.1). An asterisk indicates a significant fold change between the time points within each sampling period (Bonferroni adjusted *p*-value < 0.001).

Other transcripts annotated as proteins that play a role in cold tolerance included those for a trehalose receptor that increased 14-fold, and for several heat shock proteins. For example, two heat shock protein transcripts and a heat shock transcription factor transcript that increased between September and November, significantly decreased during the spring (Fig. 3). A significant change in glutathione-S-transferase (Fig. 4) and cytochromes P450 (Table 4) also occur for larvae in the autumn and spring.

**Table 4:** P450 table. Fold changes and adjusted *p*-values for cytochromes P450 that increased in the autumn sampling period (transcript expression levels between September and November) and then either decreased or did not change through the winter (November and March) and spring (March and May) sampling periods. An asterisk indicates a significant difference (Bonferroni adjusted *p*-value < 0.001) between time points within the sampling period. Shaded rows indicate transcripts that increased significantly in the autumn and decreased significantly the in the spring.

		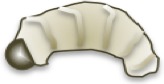	Autumn	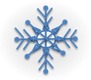	Winter	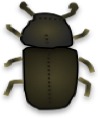	Spring	
NCBI accession	CYP ID	Fold change	Adjusted *p*-value	Fold change	Adjusted *p*-value	Fold change	Adjusted *p*-value
KB741217.1	CYP6CR2	8.01*	<0.0001	–2.79	0.0055	1.25	0.2165
KB741284.1	CYP345E3	4.86*	<0.0001	–1.85	0.1206	–4.15*	<0.0001
KB740988.1	CYP4G56	3.89*	<0.0001	1.00	1.0000	–0.60	0.3440
KB739995.1	CYP4EX1	3.29*	0.0001	–1.76	0.1901	0.04	0.9668
KB737912.1	CYP6BW2	3.16*	<0.0001	–3.98*	<0.0001	1.27	0.0678
KB740970.1	CYP9AP1	3.03*	<0.0001	–2.37	0.0016	–0.48	0.4740
KB740940.1	CYP4CV1	2.43*	0.0003	–1.13	0.9780	–0.72	0.5954
KB740092.1	CYP349B1	2.37*	<0.0001	–1.48	0.1789	–5.34*	<0.0001
KB741266.1	CYP6DF1	2.22*	<0.0001	–1.42	0.2963	–0.42	0.5217
KB740988.1	CYP9Z36	2.11*	0.0010	–3.66*	<0.0001	–2.00*	0.0004
KB741292.1	CYP6BS2	1.99*	0.0004	–1.81	0.0170	1.00	0.0543

## Discussion

As mountain pine beetle larvae grow and develop within the infested host tree, they are exposed during the autumn and winter to decreasing temperatures, conifer host chemical defenses (Bohlmann, 2012; Trapp & Croteau, 2001; Keeling & Bohlmann, 2006a.; Keeling & Bohlmann, 2006b), and pathogens (Winder, Macey & Cortese, 2010). In addition, overwintering mountain pine beetle larvae may be exposed to other possible changes in their environment such as nutrient and moisture content of host tissue or changing microbiomes of the host tissue and the feeding galleries. Many of the major fold changes in the larvae transcriptome observed between September and November occur in a group of transcripts that can be broadly grouped as “stress response genes.” Because this is a period in which mountain pine beetle larvae have shown the highest mortality (Bale, 2002), the general working hypothesis is that larvae die if they are not fully acclimated, including the production anti-freeze compounds. Our results support this, but additionally suggest that early winter larvae are under enormous physiological stress from other factors as well, such as, for example, host chemical defenses and pathogens. The autumn transcriptome signature is consistent with proteomic evidence of an increase in oxidative stress in overwintering larvae between September and November (Bonnett et al., 2012). Within this response however, it is difficult to separate the response to a seasonal drop in temperature from the other environmental pressures occurring at this time. The diversity of enzyme groups and pathways that are up-regulated between September and November indicates a multi-pronged approach to cold hardiness and overwintering. We identified several groups of genes likely to be associated with one or more “stress” factors: cold hardiness (heat shock proteins, glycerol biosynthesis, sugars and animo acids), immune-responsive proteins, and detoxification mechanisms.

Between September and November, two heat shock protein (Hsp) transcripts are strongly up-regulated (annotated as similar *to Hsp68* in *Tribolium cataneum*), an additional two transcripts are significantly up-regulated to a lesser extent (one 90kDa protein and one small heat shock protein 21), and a heat shock protein transcription factor transcript is significantly up-regulated as well. Heat shock proteins are associated with abiotic stresses such as temperature change, drought, dehydration, and chemical exposure (King & MacRae, 2015; Zhao & Jones, 2012)—all of which occur during the early months of larval growth and development under the bark of the host tree. These transcripts likely play a role in protein stabilization or sequestration in preparation for below-freezing temperatures during the winter months. Similar expression of heat shock proteins has been shown in *Drosophila melanogaster*, specifically expression of *Hsp68* was induced by acute cold stress. (Colinet & Hoffmann, 2012; Colinet, Lee & Hoffmann, 2010) and in leafminer species (Huang & Kang, 2007; Huang, Wang & Kang, 2009). The expression pattern of a putative HSP transcription factor, an *Hsp 68a*, and a small *Hsp 21* (a large increase in the early winter, stable transcription throughout the winter months, then a large decrease during the onset of spring) (Fig. 3), identifies these gene candidates for further study.

Glycerol is a known cryoprotectant in mountain pine beetle and is one of the few metabolites that have been shown to accumulate in overwintering larvae (Bentz & Mullins, 1999). Our results support the importance of glycerol production in early winter and support similar quantitative RT-PCR results found in overwintering larvae in Fraser (2011) and J Fraser (2010, unpublished data); studies that were conducted using overwintering larvae collected concurrently with the larvae sampled for this study. Key enzymes in the two catalytic steps preceding the final synthesis of glycerol were significantly up-regulated between September and November (Fig. 2). This suggests that production of glycerol is increased between September and November, in agreement with previous work (Fraser, 2011). Also in agreement with Fraser (2011), our results further suggest that glycerol is biosynthesized via dihydroxyacetone phosphate (DHAP) and glycerol-3-phosphate (G3P) rather than via glyceraldehyde (Fig. 2). Although the glyceraldehyde route could conceivably also be responsible for increased glycerol synthesis in some circumstances, we did not find evidence for this pathway in our data set, which supports the findings in Fraser (2011) as well.

Although a protein identified as trehalose-phosphate synthase was up-regulated in the autumn and down-regulated in the spring in the proteomic data reported by Bonnett et al. (2012), only a single gene transcript in our data set was annotated as a trehalose 6-phosphate synthase isoform. The expression of this transcript did not change significantly in the autumn, through the winter, or in the spring. Rozsypal et al. (2013) also found that levels of trehalose and proline remain at high, constant levels for overwintering codling moth larvae and suggest that these metabolites are important for protein and membrane stabilization during bouts of freezing temperatures as described in Jain & Roy (2009). Our data suggests a similar pattern for mountain pine beetle overwintering larvae.

In addition to preparation for sub-freezing temperatures, mountain pine beetle larvae also showed a stress response in the form of the expression of immune response transcripts. The largest significant transcript increase between September and November was annotated as a galactose-specific C-type lectin. In *Drosophila*, these enzymes are associated with immune response, but the precise role of specific proteins in this large group remains unclear (Tanji, Ohashi-Kobayashi & Natori, 2006). We identified 11 gene models in the mountain pine beetle genome annotated as ‘lectins’; two of these were highly up-regulated in larvae between September and November. This could represent either a local response to a particular pathogen, or a general stress-induced response as a result of toxic conditions of a colonized host tree.

In autumn, several genes associated with detoxification of host metabolites are also strongly up-regulated. Glutathione S-transferases and an alpha-esterases are two enzyme groups that have been implicated with detoxification of xenobiotics, such as host defense compounds, in insects (Pitt et al., 2014; Robert et al., 2013; Campbell et al., 2003; Kostaropoulos et al., 2001). As insect larvae are still living and growing in a dying tree, they would be exposed to not only decreasing temperatures, but also to the full suite of conifer host chemical defenses (Clark, Huber & Carroll, 2012; Bohlmann, 2012; Keeling & Bohlmann, 2006a.; Keeling & Bohlmann, 2006b). A large group of enzymes commonly associated with detoxification of plant defense compounds, cytochromes P450 (Feyereisen, 1999), are also significantly up-regulated in our data set during autumn. Eleven gene transcripts annotated as cytochromes P450 increase significantly in the autumn. Of these 11, three also significantly decrease in the spring (CYP345E3, CYP349B1, and CYP9Z36). These enzymes would be strong candidates for a role in larval detoxification of host tissue. CYP345E3 is similar in sequence to CYP345E2, which modifies monoterpene substrates (Keeling et al., 2013). Expressed sequence tag data suggests CYP345E3 is not localized to the antenna like CYP345E2, but found in the midgut and fat body tissues of adults (Keeling et al., 2016), also suggesting a role in detoxification. Interestingly, several of the cytochromes P450 that increased significantly in larvae during autumn (CYP6CR2, CYP4EX1, CYP9AP1, and CYP6BW2) comprise almost the entire list of significantly decreasing cytochrome P450s in starved versus fed adult mountain pine beetle (Robert et al., 2013). Because these transcripts do not decrease in spring, are elevated in antennae-rich mountain pine beetle expressed sequence tag libraries (Keeling et al., 2012), and implicated in odourant degradation processes in adult cotton leafworms (Pottier et al., 2012), it may be possible that the development of odour processing begins as early as the formation of eye-antennal imaginal discs in early instar larvae (Haynie & Bryant, 2005). In addition, CYP6CR2 has high sequence similarity to CYP6CR1, which is an epoxidase in exo-brevicomin biosynthesis, a pheromone produced by male mountain pine beetle after leaving the brood tree, but that decreases during host tree selection and mating (Song et al., 2014). Theses cytochromes P450 could also play a role as the developing larvae navigate and feed on host tissue containing a diversity of associated fungi that can act as a food source, or threat, under the bark (Lee, Kim & Breuil, 2006). The opposite process occurs in the adults, as after host colonization, odorant detection and processing becomes irrelevant as the focus shifts to mating and reproduction (Robert et al., 2013).

In spring, larvae must begin the physiological process of preparing for emergence, dispersal flight, and a chance of reproduction during the subsequent summer months as well as a number of unidentified processes that require further study. For larvae that survived the winter, the late-instar preparation for pupation shows decreased levels of stress response gene transcripts and increases in developmental gene transcripts generally. The various transcripts showing greater than 100-fold increases March and May are largely without known functional annotation in current databases. Developmental processes of overwintering beetles in the spring thus seem to represent a largely unknown set of processes that need further investigation. The function of the *pol* in *Hydra spp*. polyps is unknown, but possible functions include control of osmotic pressure, secretion of this protein to form an outer protective layer for the polyp, secretion as a component of the extracellular matrix, secretion as an anti-bacterial defense, or secretion as a signaling molecule (Aerne et al., 1996). The distant phylogenetic relationship of cnidarians to beetles make it difficult to predict potential function. Because this transcript is strongly and significantly up-regulated (greater than 200-fold increase in expression) between March and May, the protein product could play a significant role in bark beetle late-stage developmental processes.

## Conclusions

We suggest a multipronged physiological response from larvae in a toxic and cold environment during autumn. This response can be broadly characterized by gene transcripts involved in insect immune responses and detoxification, which are strongly up-regulated in the autumn and occurs within a background of preparation for winter via the production of glycerol, heat shock proteins, trehalose and other known strategies to tolerant below-freezing temperatures. In the spring, although gene transcripts associated with developmental process are present, the bulk of the transcripts are not yet associated with known gene functions.

## Additional Information and Declarations

### DNA Deposition

The following information was supplied regarding the deposition of DNA sequences: The raw RNA-seq data is available at the National Center for Biotechnology Information Sequence Read Archive (NCBI SRA) database (accession numbers: SRX1043704–SRX1043719) under the TRIA umbrella BioProject (PRJNA169907) that identifies aggregated research project data generated from the TRIA research collaborations on mountain pine beetle systems genomics.

### Data Availability

The following information was supplied regarding data availability: The DESeq output is publicly available via Figshare accessible from the following links: Sept–Nov DOI: 10.6084/m9.figshare.815969
URL: https://figshare.com/s/1031731941b18df7121a
Sept–May DOI: 10.6084/m9.figshare.815968
URL: https://figshare.com/s/5c3490cb9f906604b0cd
Sept–March DOI: 10.6084/m9.figshare.815967
URL: https://figshare.com/s/4d0d34c9445a6bc52422
March–May DOI: 10.6084/m9.figshare.815966
URL: https://figshare.com/s/a3621bce669faf301aa1.
